# Evaluation of features to support safety and quality in general practice clinical software

**DOI:** 10.1186/1472-6947-11-27

**Published:** 2011-05-03

**Authors:** Michelle Sweidan, Margaret Williamson, James F Reeve, Ken Harvey, Jennifer A O'Neill, Peter Schattner, Teri Snowdon

**Affiliations:** 1 NPS: Better choices, Better health, Level 7, 418A Elizabeth St, Surry Hills NSW 2010, Australia; 2 School of Public Health, La Trobe University, Bundoora VIC 3086, Australia; 3 Medical Software Industry Association Inc, PO Box 1293, Wahroonga NSW 2076, Australia; 4 Department of General Practice, School of Primary Health Care, Monash University, Notting Hill VIC 3168, Australia; 5 Royal Australian College of General Practitioners, 1 Palmerston Cres, South Melbourne VIC 3205, Australia

## Abstract

**Background:**

Electronic prescribing is now the norm in many countries. We wished to find out if clinical software systems used by general practitioners in Australia include features (functional capabilities and other characteristics) that facilitate improved patient safety and care, with a focus on quality use of medicines.

**Methods:**

Seven clinical software systems used in general practice were evaluated. Fifty software features that were previously rated as likely to have a high impact on safety and/or quality of care in general practice were tested and are reported here.

**Results:**

The range of results for the implementation of 50 features across the 7 clinical software systems was as follows: 17-31 features (34-62%) were fully implemented, 9-13 (18-26%) partially implemented, and 9-20 (18-40%) not implemented. Key findings included: Access to evidence based drug and therapeutic information was limited. Decision support for prescribing was available but varied markedly between systems. During prescribing there was potential for medicine mis-selection in some systems, and linking a medicine with its indication was optional. The definition of 'current medicines' versus 'past medicines' was not always clear. There were limited resources for patients, and some medicines lists for patients were suboptimal. Results were provided to the software vendors, who were keen to improve their systems.

**Conclusions:**

The clinical systems tested lack some of the features expected to support patient safety and quality of care. Standards and certification for clinical software would ensure that safety features are present and that there is a minimum level of clinical functionality that clinicians could expect to find in any system.

## Background

Electronic prescribing (e-prescribing) offers an opportunity to improve the quality, safety and efficiency of health care and is now the norm in many countries[1]. There is evidence to show that e-prescribing (often with clinical decision support) is associated with a reduction in medication errors and incomplete or unclear orders, improved drug allergy detection and greater adherence with clinical practice guidelines[2-4]. There are however also reports of unintended negative consequences of e-prescribing, for example unfavourable effects on workflow and the introduction of new types of errors[5,6].

Software systems for e-prescribing have been available for two decades, however standards and certification processes for these systems have lagged behind development and use of the software. Currently there is little information for funders or users of these systems to assess how effectively a system supports healthcare safety and quality.

In Australia, general practitioners (GPs) have been using clinical software systems that include e-prescribing for more than 15 years, with rapid uptake encouraged by government incentives in the 1990s. These systems have been developed in an unregulated environment, with little evaluation of their impact on clinical practice or health outcomes. We wished to find out if current systems include features that facilitate improved patient safety and care, with a focus on quality use of medicines. Quality use of medicines is the judicious, effective and safe use of medicines, and in terms of clinical software functionality it encompasses the entire medication management process.

In previous work we identified a list of desirable software features (where feature includes functionality or other characteristic of a software system), with each feature being rated for its expected impact on safety, quality and usefulness to the clinician and the patient[7]. In this study the features were tested in seven clinical software systems. Here we report on the software testing process and the results for a subset of the features.

## Methods

The study protocol was approved by the Australian Government Department of Health and Ageing Ethics Committee.

### Features tested

Of the 114 software features previously identified, 104 were tested in software (10 were classified as 'aspirational')[7]. In this paper we report on the results for the 50 features that were rated as likely to have a high impact on safety and/or quality of care, and that could be tested in software.

### Software systems

Seven commonly used general practice clinical systems were evaluated: Best Practice™, Genie™, Medical Director 2™, Medical Director 3™, MedTech32™, Practix™ and Profile™. Added together, the users of these systems account for more than 90% of Australian GPs. All the systems integrate prescribing within an electronic health record. For testing, the systems were purchased and installed on two dedicated laptop computers. Default settings in the software were not altered unless the test script specified a change. The software version tested was the most recent version of the software produced and available on 1 January 2008.

### Development of test scripts

**Figure 1:**
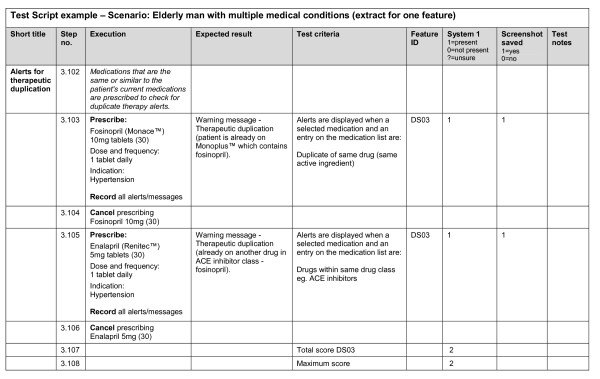
Extract from a test script.

Test scripts were developed using clinical scenarios based on 11 imaginary patients visiting their GPs. For each of the features to be tested, one or more test criteria were developed. Each test script was prepared in a separate Excel worksheet and was designed to produce a logical flow for testing and to facilitate data entry; there were 660 executable steps and 350 test criteria in total for the 104 features. The test scripts were reviewed by a GP and a health informatician--they provided feedback on the content, format and clarity of the scripts. An extract from one of the test scripts is shown in Figure 1.

### Software testing

Testing was undertaken independently by two researchers in January to April 2008. The same researchers also carried out pilot testing to assess the usability of the test scripts and how the process flowed. Full testing followed, with the process being completed for each script across all seven systems prior to starting the next script. Approximately 2000 screenshots were saved by each tester.

At the completion of testing for each script, the results were reviewed and compared between the two researchers. Where their scores were in agreement, the test results were accepted. Where there was disagreement, four researchers (ZA, AS, MS, MW) met to discuss the variation in results and reach a consensus decision, which in some cases required retesting. At the end of the test process, one of the researchers (MS) undertook a line-by-line review of all results to check the scores and to identify any results that were unclear, unexpected or inconsistent, and that required verification with an expert user or the software vendor.

A number of features could not be tested adequately within the test environment. A GP expert user of each system (nominated by the vendor of that system) was contacted in February 2009 to find out about implementation of these features in their system. The test scripts were recast as interview questions that required a yes/no response. A researcher interviewed each GP at their practice, requesting a demonstration on the computer or further details where applicable. The responses were recorded by hand. Expert users provided information on the processes for receipt of pathology results and incorporation of these results into patient records, use of messaging, availability of system logs and ease of software updates.

### Review of preliminary results with software vendors

Test data were collated and the preliminary results were provided to each software vendor, with a request for feedback. They were informed that the preliminary results for their system would be reviewed based on their feedback, and that the scores would be revised if there was sufficient evidence to show that the system included any functionality in question. A face-to-face meeting or teleconference was held with representatives from each company in April 2009. Discussion focussed on features where the test results were unclear or where there was disagreement. The representatives provided a demonstration of the system and/or documentation of functionality to support any claims. Meeting notes were sent back to each vendor, with further comment invited by email.

### Review of results by study guidance group

Scores were revised based on vendor feedback and these were provided to the study guidance group for review and approval. In accepting or rejecting the scores, the group recommended that: (1) Software features should be easily accessed by the average GP, and a system should not 'pass' where a feature is available but which requires extensive configuration or assistance from the vendor in order to use it; (2) if a feature is dependent on an external source of information (eg, many of the decision support features), it should remain as a scored feature, but with a note that the feature is dependent on the availability of a suitable knowledge resource; and (3) for some features the results available were either not sufficient to score the item at all, or not to score it quantitatively - these were designated 'not scored' or 'description only'. Most of the recommendations were accepted and scores were increased for all seven systems, with between 9 and 18% of the scores being revised across the systems.

## Results

**Figure 2:**
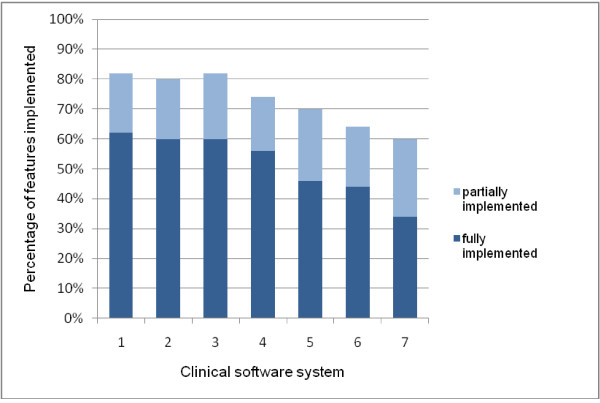
Implementation of 50 features expected to have a high positive impact on quality and safety in 7 clinical software systems.

The implementation of the 50 scored features in seven clinical software systems is shown in Figure 2 and Table 1. Figure 2 provides an aggregate view of the implementation of the features: of the 50 features, the number that were fully implemented in the 7 systems ranged from 17-31 features (34-62%), with 9-13 (18-26%) features being partially implemented, and 9-20 (18-40%) not implemented. Table 1 shows the results by individual feature. The software systems are not identified individually as our intention was to look at features across all systems and make general recommendations for improvement of clinical software.

**Table 1:** Implementation of 50 features* in 7 prescribing systems

	Feature	Clinical software system						
		
		1	2	3	4	5	6	7
	***Patient data***							

1	Create patient management plans	●	●	●	●	●	●	●

2	Record and display allergies and drug intolerance	●	●	●	●	●	●	◖

3	Record and display clinical information	●	●	●	●	◖	●	●

4	Record and display pregnancy and breast-feeding status	●	●	●	◖	◖	●	◖

5	Record, display and sort current and past medicines (other than complementary medicines)	●	◖	◖	◖	◖	◖	◖

6	Record and display patient identifying information	◖	◖	◖	◖	◖	◖	◖

7	Record complementary medicines	◖	◖	◖	◖	◖	◖	◖

8	Record medicine changes	◖	◖	◖	◖	◖	◖	◖

9	Record preventive and non-pharmacological management in a specified format	○	○	○	○	○	○	○

	***Medicine selection***							

10	Easily select correct problem or diagnosis from a list	●	●	●	●	●	●	●

11	Access to regulator-approved product information	●	●	●	●	●	●	●

12	Provide drug strength and form information	●	●	●	●	●	●	●

13	Provide adult and child dosing information	●	●	●	●	●	●	●

14	Display active ingredient/s when a branded product selected	○	●	○	●	●	○	●

15	Access to (other) evidence-based drug information at the time of prescribing	●	○	●	○	○	○	○

16	Access to dose calculators	●	○	●	○	○	○	○

17	Differentiate between similar name medicines on a list	◖	●	◖	○	○	○	○

18	Record indication for medicine prior to prescribing	○	○	○	●	○	○	○

19	Provide pre-defined dosage regimens for selection	○	○	○	○	○	○	○

	***Decision support***							

20	Alerts for drug-drug interactions	●	●	●	●	●	●	●

21	Alerts for drugs in pregnancy	●	●	●	●	●	●	●

22	Alert for same patient name (on opening or creating record)	●	●	●	●	●	●	◖

23	Alerts for drug allergy or intolerance	●	●	●	●	◖	◖	◖

24	Preview and confirm correct patient details prior to electronic transmission of data	◖	●	◖	●	◖	●	◖

25	Alerts for drugs in breastfeeding	●	●	●	○	○	○	○

26	Alerts for therapeutic duplication	●	●	●	○	○	○	○

27	Alerts for drug-condition contraindications	●	◖	●	○	○	○	○

28	Alerts for drug use in patients with renal impairment	●	○	●	○	○	○	○

29	Alerts prioritised by importance	○	○	○	●	○	●	○

30	Access to patient data related to alert	○	○	○	○	●	●	○

31	Alerts for harmful dose, dosage regimen or quantity	◖	○	◖	○	○	○	○

32	Alerts for safety issues related to specific products	○	○	○	○	○	○	○

	***Patient education and information***							

33	Provide patient clinical information and tools	●	◖	●	◖	●	◖	◖

34	Produce current medicine list--patient version	◖	●	◖	◖	◖	◖	◖

35	Provide patient medicine information	◖	◖	◖	◖	◖	◖	◖

	***Monitoring, reminders and recalls***							

36	Reminder--action test results	●	●	●	●	●	●	●

37	Reminder--overdue tests	●	●	●	●	●	●	●

38	Displays laboratory results usefully	●	●	●	●	●	●	●

39	Recalls--manually flagged for individual patients	●	●	●	●	●	●	●

40	Produce reports--individual and practice level	●	●	●	●	◖	●	●

41	Display medicine information with relevant outcomes	●	●	●	●	●	○	○

42	Produce reports--predefined	●	●	●	◖	●	○	○

43	Produce reports--user defined	◖	◖	◖	●	◖	◖	○

44	Recalls and reminders--preventive care and public health programs	◖	◖	◖	●	○	○	○

45	Reminder--routine or recommended tests	○	◖	○	○	○	○	○

	***Interoperability and communication***							

46	Import/export of a range of file formats	●	●	●	●	●	◖	◖

47	Generate standard patient medicine chart	●	●	●	●	●	○	○

	***Security and administration***							

48	System back-up and restore process is straightforward and secure	●	●	●	●	●	●	●

49	System and content updates are reliable and easy to install	●	●	●	●	●	●	●

	***Transparency ***							

50	Exclude all advertising	Advertising was present in 2 systems**						

● fully implemented (all test criteria met)

◖ partially implemented (some test criteria met)

○ not implemented (no test criteria met)

Notes

* There were 7 other features that were classified as high impact on safety and/or quality but are not included in this table because they were either (a) tested but not able to be scored (knowledge base--content quality; use of standard messaging protocol; import/export patient clinical data) or (b) were considered 'aspirational' at the time of the study and were not tested (alerts for best practice therapy; use of standard clinical terminologies and coding; support for selection of therapeutic options; use of a national unique patient identifier).

** Not shown as this would enable identification of these systems.

Important safety features that were included in all or most systems were alerts for drug-drug interactions, drugs in pregnancy and allergies. Most systems displayed information about allergies and pregnancy and breastfeeding status throughout the consultation. All systems had reminders for new pathology results that were abnormal and for overdue pap smears, and warned the user when creating or opening a record where there was another patient with the same name in the system.

There were also some gaps and limitations, as follows:

*• Variable decision support for prescribing*. Some systems provided decision support for therapeutic duplication (3 systems), drug-condition contraindications (3 systems), drug use in breastfeeding (3 systems) and renal impairment (2 systems). There was little or no decision support for harmful dosage regimens or for safety issues related to specific products, such as recent warnings issued by the Therapeutic Goods Administration. Several software vendors cited lack of viable access to suitable knowledge bases to implement some of these features.

*• Limited access to evidence-based drug and therapeutic information*. No system provided access to information from either of two key Australian medicines references--the Australian Medicines Handbook and Therapeutic Guidelines. These resources provide independent, evidence based drug information but at present they are not available in a format that can readily be incorporated into clinical software systems. In relation to drug dosage, all systems provided adult and child dosing information that was based on the Australian regulator-approved product information, however this information source has limitations as it does not include off-label indications and often does not include paediatric dosage.

*• Potential for medicine mis-selection at the time of prescribing*. The display of medicines in most systems was not optimal--long lists of products were displayed in small windows that required extensive scrolling, and the same or similarly named products were listed together with no clear differentiation between them. Only one system implemented a method to reduce the risk of mis-selection by making product selection a two-step process.

*• Linking a medicine with its indication was optional*. Linking was possible in all systems but in no case mandatory. Linking is important so that other health providers know what the medicine is for when health information is communicated or shared, and for quality improvement activities eg, comparison of own prescribing versus best practice guidelines.

*• Definition of 'current medicines' vs 'past medicines' was not always clear*. Some systems moved a medicine from the 'current' to the 'past' list automatically after a certain period. This is a crucial component of a health record and there is no standard definition of 'current medicines' and how they should be handled in an electronic health record.

*• Medicines lists for patients were suboptimal*. All systems produced a patient version of the current medicines list but in most cases it was suboptimal, being poorly formatted, omitting the purpose of the medicine, or using Latin or non-standard abbreviations.

*• Some systems did not have warnings for allergy cross-sensitivities*. Three systems did not provide an alert when a cephalosporin was prescribed in a penicillin-allergic patient.

*• No standard way to record preventive care and non-pharmacological management*. This is an increasingly important aspect of care; details were able to be recorded in different parts of the record or not at all.

*• Limited patient resources*. All systems provided access to Australian consumer medicines information leaflets. Availability of other patient resources was variable, ranging from none at all to two systems with a large number of resources, including leaflets on specific medicines, health conditions and nutrition.

*• Variable clinical reporting*. The type and number of predefined clinical reports varied between systems. All systems enabled user-defined reports to be produced; however, the flexibility, sophistication and ease of use varied markedly. For example, in only one system could we easily produce a report to identify patients who had not returned for their third dose of human papillomavirus vaccine.

*• Pharmaceutical advertising was present in two systems*. This was displayed at the time of prescribing, often in a colourful, flashing format. Subsequently both systems removed this form of advertising.

## Discussion

### Findings

The seven clinical software systems tested in this study all included some important safety and quality features however there were also gaps and limitations, as outlined above. The scope and quality of decision support features were particularly variable--this followed from differences in the knowledge bases used, or in some cases differences in the way a knowledge base was incorporated in the system. Some recommended features could not be implemented at the time of the study, for example because there was no nationally accepted messaging standard or drug and disease terminology.

The user interfaces differed substantially from system to system and many features were implemented in different ways, reflecting in part a lack of guidance for standardisation of clinical software. Up until recently there was little information or evidence on how to design the clinical software interface for usability and patient safety, however there is now work being done in this area[8-10].

Many of the software vendors welcomed the feedback from the study and indicated that they would incorporate changes to improve their products. Apart from one company which develops its own in-house drug and decision support database, the others were reliant on commercial knowledge bases for decision support and they had little control over the content. They are keen for guidance and standards to facilitate development and interoperability of their products. The study has raised awareness of these issues in the medical software industry and has stimulated discussion about software standards.

### Strengths and limitations

The software evaluation process was complex and resource intensive, and has not been done on this scale before in Australia. The methods for producing test scripts and the test process itself were developed and refined over the course of the project. Limitations of the study include that we did not measure the usability of features or examine in detail the quality of the integrated information, thus features that were fully implemented were present but would not necessarily perform optimally in practice. A small number of test examples were used to test each feature; these were intended to be typical and were not exhaustive. Lastly, testing was done in a 'laboratory' environment using the default system settings; in real-life practice, these systems may perform differently depending on the local system configuration and prescriber behaviour.

### Other research

Other researchers have also reported limitations in the safety features and functionality of clinical software systems. Fernando et al tested prescribing safety features in four general practice computer systems that were used by about 75% of GP practices in the UK[11]. The number of appropriate alerts displayed in the four systems tested ranged from three to seven, out of a maximum score of 18. Examples of scenarios tested were "aspirin prescribed for a child of 8 years", "methotrexate prescribed in pregnancy" and "sildenafil prescribed to a patient already using isosorbide mononitrate". Overall, problems included a lack of contraindication alerts, the presence of spurious alerts, failure of drug allergy warnings, ease of overriding most alerts, and lack of audit trails[12].

Wang et al[13] conducted a field study in the United States to find out which of 60 recommendations to improve patient health outcomes and patients' ability to manage costs made by Bell et al[14] had been implemented in ten outpatient clinical software systems. For example, they examined whether systems provided access to evidence based information on drug effectiveness and safety at the time of prescribing, and whether the prescriber was alerted when a medication selected had a contraindication or precaution based on the patient's allergies, current medications, medical conditions or laboratory findings. There was marked variation between the ten systems tested, with implementation of the 60 recommendations ranging from 33-77%. There are similarities between our results and those of both Fernando et al[11] and Wang et al,[13] notably in the variable implementation of software features related to the medicine selection process and clinical decision support.

### General recommendations

Some general recommendations can be made based on our findings. Clinical decision support should be based on high quality, evidence based knowledge bases and appropriate rules; it should be clear and concise, and should include details about the source and currency of the information. Messages that disrupt the workflow (eg. alerts and warnings) should be limited in number to reduce alert fatigue, and information of lesser clinical importance should not interrupt the workflow. Clinical resources should be readily accessible during the consultation, with information available in a format that is easy to navigate (eg. not large PDF documents). Patient resources and reports should be presented in a user-friendly format and use appropriate language.

The system should assist the clinician to maintain up to date medicines and conditions lists. In order to reduce selection errors, pick lists (eg. for medicines) should be limited in length and should be presented so that it is easy to differentiate between similar items. Scrolling should be avoided where possible, and especially in small windows. The query and reporting function should be flexible and easy to customise by an average user. The import and export of patient data between systems would be facilitated by an agreed document structure, use of the same (or mapped) medicines and clinical terminologies, and a defined minimum dataset for general practice (or other domain).

### Need for software standards and certification

Our study shows that current Australian clinical software systems include some beneficial decision support tools and useful resources, but that there are also noteworthy flaws, such as potential for medicine selection errors and lack of or poor quality decision support in some areas. Standards and certification for clinical software would ensure that safety features are present and that there is a minimum level of clinical functionality that clinicians could expect to find in any system.

There is work underway internationally to define clinical software capabilities in various healthcare settings. For example in the UK the NHS has defined functional specifications for secondary care,[15] and data interchange standards for primary care[16]. In the US the Certification Commission for Health Information Technology (CCHIT) has produced certification criteria for a range of different types of electronic health record systems[17]. The feature list from this study could be used as the basis for development of software standards to support quality and safety in Australian general practice.

### Further work

There is a lot of activity in this area--much is being learnt and new functionality will become possible as technologies and systems are developed and software vendors seek to improve their systems. Any list of recommended software features or evaluation of software systems requires ongoing review.

From a research perspective, some of the features could be evaluated in more depth, as we have done previously for drug interaction decision support[18]. It was evident during the study that more work needs to be done to develop high quality knowledge bases that provide content for decision support, and that usability is a crucial issue that warrants evaluation and development of guidelines for software developers.

## Conclusion

Clinicians are increasingly reliant on their clinical software systems. Current systems used by general practitioners in Australia vary widely in their ability to support quality and safety in relation to prescribing and use of medicines. The work we have undertaken is an important precursor to achieving greater standardisation of clinical software systems.

## Competing interests

JO represented the Medical Software Industry Association on the study guidance group; the MSIA covers more than 80 commercial companies with products and services in health IT.

## Authors' contributions

MW, MS and JR developed the idea for the study. All authors contributed to the study design and reviewed the results. MS carried out some of the software testing, analysed the results and drafted the manuscript. All authors revised the manuscript critically and approved the final manuscript.

## Pre-publication history

The pre-publication history for this paper can be accessed here:

http://www.biomedcentral.com/1472-6947/11/27/prepub
